# Retraction: Genome-wide analysis of sucrose synthase family in soybean and their expression in response to abiotic stress and seed development

**DOI:** 10.1371/journal.pone.0274409

**Published:** 2022-09-14

**Authors:** The PLOS ONE Editors

The *PLOS ONE* Editors retract this article [1] because it was identified as one of a series of submissions for which we have concerns about authorship, competing interests, and peer review. We regret that the issues were not addressed prior to the article’s publication.

JD agreed with the retraction. MZA, HIA, AG, ZS, BA, SA, AJ, and JAN did not agree with the retraction. AAA-G, MSE, and HD either did not respond directly or could not be reached.
